# Dantrolene use across surgical and medical care at Mayo Clinic from 2010 to 2024: Indications, frequency, and value for identifying malignant hyperthermia

**DOI:** 10.17305/bb.2025.13340

**Published:** 2025-11-17

**Authors:** David Cho, Toby N Weingarten, Juraj Sprung, Aurelia Zodl, Martin J Ryll, Tracy E Harrison

**Affiliations:** 1 Mayo Clinic Alix School of Medicine, Rochester, MN, USA; 2 Department of Anesthesiology and Perioperative Medicine, Mayo Clinic College of Medicine and Science, Rochester, MN, USA

**Keywords:** Dantrolene, malignant hyperthermia, surrogate marker, general anesthesia

## Abstract

Dantrolene is the definitive treatment for malignant hyperthermia (MH), a rare and life-threatening disorder. This retrospective study aimed to achieve two objectives: (1) to characterize the indications and frequency of dantrolene administration in both medical and surgical settings and (2) to evaluate whether perioperative dantrolene may serve as a surrogate marker for identifying MH cases. Using pharmacy records, we identified hospitalized patients who received dantrolene between 2010 and 2024. Each recipient underwent a chart review to examine the clinical context of dantrolene administration. A total of 1,199,450 inpatient pharmacy records were reviewed, revealing 118 patients who received dantrolene, resulting in an incidence rate of 1 in 10,165 hospital admissions (95% CI: 1 in 8,488 to 1 in 12,280). Among these, 87 patients (74%) received oral dantrolene: 84 for chronic spasticity, two for neuroleptic malignant syndrome, and one as preoperative prophylaxis due to a history of MH. The remaining 31 patients (26%) received intravenous dantrolene. Seventeen patients received perioperative dantrolene for suspected MH; of these, nine cases (53%) were subsequently clinically confirmed as MH. Based on these findings and the total number of surgical procedures involving general anesthesia (*n* ═ 885,127), the estimated prevalence of MH following general anesthesia was calculated to be 1 in 98,328 exposures (95% CI: 1 in 51,813 to 1 in 215,054). Dantrolene was administered at an approximate rate of 1 per 10,000 hospital admissions, primarily in oral formulation for chronic spasticity. Among the patients who received perioperative dantrolene, approximately half were confirmed to have MH, resulting in an estimated MH prevalence of 1 in 100,000 patients exposed to general anesthesia.

## Introduction

Dantrolene, a direct-acting skeletal muscle relaxant, is rarely utilized in general clinical practice. It is occasionally prescribed for conditions associated with muscle spasticity, including spinal cord injury, stroke, multiple sclerosis, and cerebral palsy. Moreover, dantrolene is critical in managing several emergent conditions, such as malignant hyperthermia (MH), neuroleptic malignant syndrome (NMS), tetanus, serotonin syndrome, heat stroke, and catecholaminergic polymorphic ventricular tachycardia. In the surgical context, dantrolene is recognized as the specific first-line treatment for MH.

MH is a rare, life-threatening hypermetabolic reaction that typically occurs in the perioperative setting and follows an autosomal dominant inheritance pattern. This condition is triggered by uncontrolled calcium (Ca^2+^) release from the sarcoplasmic reticulum (SR) due to mutations in the ryanodine receptor type 1 (RYR1) Ca^2+^ channel [1]. The RYR1 receptor is located on the SR membrane of skeletal muscle cells. In individuals with MH susceptibility (i.e., those carrying mutations in the *RYR1* gene), a severe hyperthermic crisis can be induced by exposure to volatile anesthetic agents (such as sevoflurane, desflurane, and isoflurane) or depolarizing muscle relaxants like succinylcholine. In these patients, the mutated RYR1 Ca^2+^ channel remains open, resulting in excessive Ca^2+^ release into the cytosol of muscle cells. This Ca^2+^ overload leads to sustained skeletal muscle contraction (muscle rigidity), myofibrillar disruption (rhabdomyolysis), increased carbon dioxide production (hypercapnia and respiratory acidosis), metabolic acidosis, and potentially fatal hyperthermia. By inhibiting Ca^2+^ release through the RYR1 channel, dantrolene mitigates cross-bridge formation between actin and myosin, reduces skeletal muscle contraction, and alleviates the hypermetabolic crisis.

Intravenous (IV) dantrolene is typically administered in urgent clinical situations. Given its specificity for MH and limited alternative applications, its administration in the perioperative setting can serve as a proxy indicator for MH [2, 3]. This study aims to review the pharmacy records from the Mayo Clinic Rochester over a 14.5-year period to identify patients who received either IV or peroral dantrolene (PO), representing a diverse patient population across both surgical and medical settings. Our primary objective is to assess the frequency and clinical indications for dantrolene use among hospitalized patients and to evaluate the accuracy of perioperative IV dantrolene in identifying MH, thereby estimating its prevalence in the perioperative context.

## Materials and methods

This study was approved by the Mayo Clinic Institutional Review Board (identification number: 24-007824, approved October 7, 2024). In accordance with Minnesota Statute 144.295, patients who declined prior written authorization for the use of their medical records in research were excluded from this study. This manuscript adheres to the applicable STrengthening the Reporting of OBservational studies in Epidemiology (STROBE) guidelines [4].

### Study design

This retrospective study was conducted at the Mayo Clinic Hospital in Rochester, Minnesota, a quaternary academic medical center. Each hospital admission generates a corresponding pharmacy record; thus, the number of pharmacy records corresponds to the total number of hospital admissions (both inpatient and outpatient). We reviewed pharmacy records for inpatients from January 1, 2010 to June 30, 2024 to identify patients who received dantrolene. Concurrently, data from the Mayo Clinic Anesthesia Database were utilized to determine the number of patients who received general anesthesia, regional anesthesia, and monitored anesthesia care for both inpatient and outpatient cases.

For patients who received dantrolene, medical records were examined to ascertain the intended indication for its use. For those who received IV dantrolene intraoperatively or in the immediate postoperative period, we assessed the type of anesthetic management and documented the anesthetic agents employed. Clinical presentations, treatments, and outcomes were analyzed further. When available, the final clinical diagnosis was recorded; if a definitive diagnosis was not established, we documented the differential diagnosis noted in the medical records. Descriptive statistics were employed for data analysis. We report the prevalence of dantrolene administration across the medical and surgical inpatient populations, with a primary focus on surgical patients to determine the prevalence of MH.

## Results

### Indications for dantrolene administration

From January 1, 2010 to June 30, 2024, a comprehensive review of 1,199,450 pharmacy records for inpatient hospital admissions was conducted to identify patients who received dantrolene. During this period, we identified 118 patients who were administered dantrolene, yielding an estimated rate of 1 in 10,165 hospital admissions (95% CI: 1:8,488 to 1:12,280). Dantrolene was administered orally to 87 patients (73.4%); of these, 84 were treated for chronic spasticity, two for suspected NMS, and one as preoperative prophylaxis in a patient with a known history of MH. The remaining 31 patients (26.3%) received IV dantrolene: 17 in the perioperative setting and 14 outside of it (see Table S1).

### Prevalence of MH in surgical population

During the study period, 1,326,411 patients received anesthetic care from our department for both inpatient and outpatient procedures. Among these, 885,127 underwent general anesthesia, 76,282 received regional anesthesia, and 365,002 experienced monitored anesthetic care. Seventeen patients were administered IV dantrolene in the perioperative setting, of which nine were diagnosed with MH, all linked to triggering agents during general anesthesia (see Table 1). Four of these nine patients underwent genetic testing, which confirmed the presence of a *RYR1* variant associated with MH susceptibility.

**Table 1:** Use of dantrolene in hospitalized patients with suspected malignant hyperthermia

**Age** **Sex**	**Procedure** **triggering agents**	**Clinical course,** **therapies, outcomes**	**MH diagnosis** **genetic testing and/or clinical**
20 F	Oral cyst excision SUX, DES	Masseter spasm with induction, unremarkable anesthetic course for 1-h procedure. During emergence increase in EtCO_2_ 55 mmHg with respiratory rate to 50 per minute. pH 7.32, PaCO_2_ 43 mmHg, BE --4 mmol/L, K 4.1 mmol/L. Remained afebrile. Dantrolene (2.5 mg/kg during surgery) administration, hydration. Complaint of lower extremity muscle soreness several hours. CK_max_ 11,502 U/L. Full recovery. Past Anest Hx, *n* ═ 13: ASD repair (ISO); Liver Tx x2 (ISO, SUX); GI procedures x6 (SEVO, DES); Pectus excavatum repair (ISO); Minor procedures x3 (SEVO)	MH – confirmed Heterozygous RYR1 mutation c.7354C>T (p.Arg2452Trp)
12 M	Cardiac catheterization SEVO	90 min after induction abrupt development of fever (38.8 ^∘^C), EtCO_2_ 74 mmHg, pH 7.16, PaCO_2_ 76 mmHg, BE --7 mmol/L, K 6.3 mmol/L. Non-triggering anesthetic, dantrolene (2.5 mg/kg during surgery followed by 24-h infusion), cooling, hydration. Vital signs normalized. Remained intubated for 24 h. CK_max_ 538 U/L, urine myoglobin 30 mcg/L. Full recovery. Past Anest Hx *n* ═ 4: Heart operations x2 (ISO, SEVO); Minor procedures x2 (ISO, SEVO).	MH – confirmed Heterozygous RYR1 mutation c.6617C>T (p.Thr2206Met)
7 M	Tympanomastoidectomy SEVO	5-h after induction rapid development of tachycardia (HR 170), tachypnea, hypotension, fever (39.0 ^∘^C), EtCO_2_ 100 mmHg, pH 7.04, PaCO_2_ 78 mmHg, BE --9 mmol/L, K 6.6 mmol/L. Non-triggering anesthetic, dantrolene (five 2.0 mg/kg doses over 1 h during surgery), cooling, hydration. Vital signs normalized. CK_max_ 6,694 U/L, lactate 6.3 mmol/L, urine myoglobin 64 mcg/L. Full recovery. Past Anesth Hx, *n* ═ 2: Tympanostomy (SEVO); Myringotomy (SEVO)	MH – confirmed Heterozygous RYR1 mutation c.7300G>A
33 M	Retroperitoneal mass resection ISO Serotonergic medications: cannabis, sertraline, fentanyl	Gradual increase in temperature (38.4 ^∘^C) and EtCO_2_ 53 mmHg over 11-h surgery with intraoperative pH 7.24, PaCO_2_ 51 mmHg, BE --6 mmol/L. Initial diagnosis was serotonin syndrome treated with cooling, cyproheptadine. Because of persistent fevers (39.5 ^∘^C) dantrolene administered 24-h after surgery (100 mg doses every 5 h for 3 doses). At time of dantrolene administration pH 7.32, PaCO_2_ 55 mmHg, BE --6 mmol/L. CK_max_ 32,910 U/L, lactate, K 6.1 mmol/L, urine myoglobin 850 mcg/L, ALT 235 U/L, AST 865 U/L. Full recovery. Past Anesth Hx: TIVA x2	MH – confirmed Heterozygous RYRI mutation c.6617 C>T (p.Thr2206Met)
60 M	Carotid endarterectomy SUX, ISO	3-h after induction rapid increase in EtCO_2_ 100 mmHg, temp 40.8 ^∘^C, pH 7.14, PaCO_2_ 80 mmHg, BE --2 mmol/L, K 6.0 mmol/L. Non-triggering anesthetic, dantrolene (150 mg during surgery), cooling with temp and EtCO_2_ normalizing < 30 min, hydration. CK_max_ 3,544 U/L, urine myoglobin 378 mcg/L. Full recovery within 24 h. Past Anesth Hx: TIVA x1	MH – confirmed
74 F	Lumbar spine surgery SUX, ISO	Unremarkable for 6-h case. PACU: severe full body muscle rigidity, fever (39.4 ^∘^C), SpO_2_ 88%, tachypneic, mental status changes, pH 7.08, PaCO_2_ 82 mmHg, BE --6 mmol/L, K 4.9 mmol/L, CK_max_ 1,095 U/L. Dantrolene (220 mg during surgery, 70 mg every 6 h for 24 h), cooling, hydration. Intubated for 24-h with full recovery. Past Anesth Hx: No	MH – confirmed
61 F	Breast surgery SUX, ISO	2-h after induction rapid increase in EtCO_2_ 61 mmHg, fever (38.4 ^∘^C), pH 7.25, PaCO_2_ 59 mmHg, BE --2 mmol/L, K 6.0 mmol/L. Surgery terminated, dantrolene (180 mg during surgery, 100 mg IV every 6 h for 24 h, followed by 100 mg oral every 8 h for 3 days), cooling, hydration. CK_max_ 5,962 U/L, urine myoglobin 1,250 mcg/L. Full recovery. Past Anesth Hx: No	MH – confirmed
3-month F	MRI SEVO, ISO	Induction with SEVO switched to ISO. With 6 min rapid increase in EtCO_2_ 89 mmHg. Non-triggering anesthetic, dantrolene (10 mg during procedure), hydration. T_max_ (36.7 ^∘^C), pH 7.07, PaCO_2_ 72 mmHg, BE --9 mmol/L, K 4.0 mmol/L. Full recovery. Past Anesth Hx: No	MH – confirmed
38 M	Hip arthroscopy SEVO	During emergence after a 6-h surgery: muscle rigidity, tachycardia, afebrile, metabolic acidosis: pH 7.14, PaCO_2_ 53 mmHg, BE --10 mmol/L, K 4.9 mmol/L. Dantrolene (2.5 mg/kg during surgery), hydration, reintubated/mechanical ventilation. CK_max_ 796 U/L. Full recovery. Past Anesth Hx: Arthroscopy x2 (SUX, SEVO, ISO); Laminectomy (SUX, SEVO, ISO)	MH – confirmed

Abbreviations: M: Male; F: Female; MH: Malignant hyperthermia; SUX: Succinylcholine; DES: Desflurane; EtCO_2_: End-tidal carbon dioxide; BE: Base excess; CK_max_: Maximum creatine kinase; RYR1: Ryanodine receptor type 1; SEVO: Sevoflurane; ISO: Isoflurane; IV: Intravenous; ALT: Alanine aminotransferase; AST: Aspartate aminotransferase; PACU: Postanesthesia care unit; MRI: Magnetic resonance imaging; NMS: Neuroleptic malignant syndrome; ECT: Electroconvulsive therapy; TIVA: Total intravenous anesthesia; Past Anest Hx: Indicates exposure to general anesthesia before currently reported events. Reference values: CK, reference, 39–308 U/L; ALT, reference, 7–55 U/L; AST, reference, 8–48 U/L; Urine myoglobin, reference, ≤65 mg/L.

Additionally, eight patients exhibited an MH-like clinical presentation but were subsequently diagnosed with alternative conditions, including sepsis, NMS, or serotonin syndrome (see Table 2). Based on the confirmed cases of MH, the estimated prevalence of this condition was 1 in 98,347 exposures to general anesthesia (95% CI: 1:51,808 to 1:215,078). Notably, four patients with MH had multiple prior uneventful exposures to volatile anesthetics, two had previously undergone uneventful procedures under total IV anesthesia, and three had no prior history of anesthesia exposure.

**Table 2:** Hospitalized patients treated with dantrolene for suspected malignant hyperthermia later diagnosed with alternative conditions

**Age** **Sex**	**Procedure** **triggering agents**	**Clinical course,** **therapies, outcomes**	**Final** **diagnosis**
63 F	Cardiac surgery SUX, ISO Serotonergic medications: methylene blue, sufentanil, omeprazole, venlafaxine	8-h after surgery in ICU developed respiratory acidosis, fever, ectopy, hyperdynamic cardiac index (11 LPM), muscle rigidity, pH 7.24, PaCO_2_ 55 mmHg, BE --4 mmol/L, K 6.4 mmol/L. Dantrolene, cooling, diuresis with resolution of symptoms within 6 h. CK_max_ 2,179 U/L. Full recovery except perioperative stroke.	Final diagnosis not established, either serotonin syndrome or NMS
45 M	Cardiac surgery ISO	Persistent fever (39.7 ^∘^C) for 24-h after surgery, pH 7.39, PaCO_2_ 47 mmHg, BE +3 mmol/L, CK_max_ 1,625 U/L, K 6.1 mmol/L. Empiric dantrolene, cooling. Full recovery.	Final diagnosis not established
88 F	ECT, SUX Serotonergic meds: fluoxetine, omeprazole	After ECT became rigid, febrile (39.2 ^∘^C), pH 7.46, PaCO_2_ 35 mmHg, BE +1 mmol/L. Empiric dantrolene, cooling, cyproheptadine. CK_max_ 47 U/L. Full recovery.	Final diagnosis not established, either serotonin syndrome or NMS
39 M	Cardiac transplant, ISO, SUX	Day of surgery in ICU developed fever (41.7 ^∘^C) diffuse shivering, pH 7.37, PaCO_2_ 47 mmHg, BE +1 mmol/L. 14-h after surgery, dantrolene empirically initiated. The fever resolved within 24 h.	Sepsis and multiorgan failure
65 F	Total hip arthroplasty SEVO, SUX	6-h after surgery developed tachypnea, hypotension, diaphoresis, fever (40 ^∘^C). pH 6.98, PaCO_2_ 22 mmHg, BE --26 mmol/L, lactate 16 mmol/L, CK_max_ 235 U/L, urine myoglobin > 5000 mcg/L, AST 22,620 U/L, INR 7.9. Empiric dantrolene without improvement. Died postoperative day 2 of multiorgan failure.	Sepsis and multiorgan failure
66 M	Cardiac surgery Non-triggering anesthetic	Had chronically elevated CK, so non-trigger anesthetic was used. 8-h after surgery became febrile (39.3 ^∘^C). pH 7.24, PaCO_2_ 44 mmHg, BE --8 mmol/L, K 4.2 mmol/L. Administered dantrolene with resolution of fever. CK_max_ 1,545 U/L. Positive blood cultures, given antibiotics. Full recovery.	Sepsis
61 Y	Abdominal exploration ISO	Aortic root replacement for infective endocarditis. Next day an emergent abdominal exploration underwent. He had fever (41.0 ^∘^C), pH 7.33, PaCO_2_ 38 mmHg, BE --6 mmol/L, K 6.5 mmol/L. Empiric dantrolene administered though suspicion of MH was low. No clinical improvement. Died two days later of multiorgan failure.	Sepsis
53 M	Intubated for hemodynamic instability SUX	Hospitalized for dysrhythmia and ICD discharge in setting congenital heart disease and acute heart failure. Intubated on hospital day 1 for hemodynamic instability with SUX. Developed fever (40.9 ^∘^C) and was administered single dose dantrolene without improvement. pH 7.35, PaCO_2_ 42 mmHg, BE --2 mmol/L, K 4.7 mmol/L, CK_max_ 194 U/L, urine myoglobin 68 mcg/L. Died hospital day 8 from sepsis and multiorgan failure.	Sepsis

Abbreviations: M: Male; F: Female; MH: Malignant hyperthermia; SUX: Succinylcholine; EtCO_2_: End-tidal carbon dioxide; BE: Base excess; CKmax: Maximum creatine kinase; SEVO: Sevoflurane; ISO: Isoflurane; ALT: Alanine aminotransferase; AST: Aspartate aminotransferase; ICD: Internal cardiac defibrillator; PACU: Postanesthesia care unit; MRI: Magnetic resonance imaging; NMS: Neuroleptic malignant syndrome; ECT: Electroconvulsive therapy. Reference values: CK, reference, 39–308 U/L; ALT, reference, 7–55 U/L; AST, reference, 8–48 U/L; Urine myoglobin, reference, ≤65 mg/L; Serum lactate, reference 0.60–2.30 mmol/L.

### Clinical characteristics of MH cases

Among the nine patients diagnosed with MH, four underwent genetic sequencing, revealing a heterozygous mutation in the *RYR1* gene, while the remaining five were diagnosed based on clinical criteria. All nine patients received a volatile anesthetic, and four were also administered succinylcholine. Six patients experienced an abrupt onset of symptoms during general anesthesia, while one experienced symptoms during the post-anesthesia recovery period. For instance, a 33-year-old male undergoing an 11-h retroperitoneal mass resection exhibited a gradual onset of hypercarbia, hyperthermia, and metabolic acidosis over 24 postoperative hours. Prior to surgery, he was treated for depression with sertraline and received fentanyl during the procedure, leading to an initial diagnosis of serotonin syndrome and treatment with cyproheptadine. However, persistent fever prompted empirical treatment with dantrolene, resulting in clinical improvement. Subsequent genetic testing confirmed a mutation in the *RYR1* gene. Another case involved a 38-year-old male who underwent a 6-h uneventful orthopedic surgery with sevoflurane. During emergence, he developed profound muscle rigidity and hyperventilation, with tidal volumes exceeding 1 L and minute ventilation exceeding 20 L/min without elevated end-tidal carbon dioxide (EtCO_2_). Arterial blood gas analysis revealed a pH of 7.14, PaCO_2_ of 53 mmHg, and a base excess of --10 mmol/L. He received 2.5 mg/kg IV dantrolene, and a subsequent arterial blood gas analysis, conducted 90 min later, showed a pH of 7.50, PaCO_2_ of 27 mmHg, and a base excess of --2 mmol/L. He remained afebrile, with a maximum serum creatine kinase (CKmax) level of 796 U/L. Lastly, a 60-year-old man undergoing carotid endarterectomy (patient #5 in Table 1) experienced a gradual onset of hypercarbia early in the anesthetic course; however, this went unrecognized, and instead of identifying MH, attempts were made to compensate for the rising EtCO_2_ by increasing minute ventilation.

### Dantrolene administration in MH suspected cases

Of the additional eight cases (Table 2) in which MH was initially suspected and dantrolene was administered, five were attributed to sepsis and/or multi-organ failure. Importantly, all these MH-like events occurred within the perioperative setting and involved exposure to volatile anesthetics. Two cases were associated with the use of multiple serotonergic medications and were subsequently diagnosed as serotonin toxicity. The remaining case involved a 45-year-old male who underwent cardiac surgery and developed a persistent fever 24 h postoperatively. In the absence of acidosis, MH was considered unlikely; however, dantrolene was administered empirically.

### Oral dantrolene administration in NMS cases

This report presents two cases in which oral dantrolene was utilized to treat suspected NMS. The first patient, a 73-year-old female, exhibited altered mental status, rigidity, and fever (40.0 ^∘^C) following haloperidol administration for agitation. Laboratory results indicated: pH 7.42, PaCO_2_ 23 mmHg, BE --9 mmol/L, CKmax 633 U/L. She responded positively to dantrolene and hydration, resulting in a full recovery. The second patient, a 78-year-old female, was admitted for altered mental status. On the sixth day of hospitalization, following paliperidone administration, she experienced worsening sensorium, hypercarbic respiratory failure, and fever (38.8 ^∘^C). Her laboratory findings included: pH 7.39, PaCO_2_ 50 mmHg, BE +5 mmol/L, K 3.8 mmol/L, CKmax 86 U/L. Dantrolene was administered empirically for suspected NMS; however, she did not improve and was ultimately diagnosed with toxic encephalopathy, passing away 35 days later.

### Dantrolene IV use in conditions unrelated to MH

Table S1 summarizes 14 patients who received IV dantrolene without any suspicion of MH. Among these, nine patients were suspected of having NMS, with eight confirmed as such. The ninth case involved a 37-year-old female admitted with unresponsiveness and high fever. She was empirically treated with both cyproheptadine and dantrolene but was ultimately diagnosed with bacterial meningitis and died. Three patients received dantrolene for symptomatic relief of muscle rigidity: a 44-year-old male with rigidity secondary to catatonia, a 13-year-old male with trismus secondary to juvenile dermatomyositis, and a 24-year-old female with episodic dystonia due to anti-NMDA receptor autoimmune encephalitis linked to an ovarian teratoma. Additionally, a 63-year-old male with cerebral palsy and spasticity had his chronic oral dantrolene substituted with the IV formulation after developing pneumonia and sepsis, requiring intubation and mechanical ventilation. Finally, a 19-year-old male developed central fever secondary to polytrauma and was treated with oral bromocriptine for central dysautonomia. Following an abdominal operation during hospitalization, his antipyretic management was transitioned to IV dantrolene.

## Discussion

The use of dantrolene in our clinical practice was infrequent. When administered orally, it primarily serves to treat spasticity. In surgical settings, IV dantrolene is typically reserved for suspected MH cases, as treatment is critical even with minimal suspicion due to the high risk of mortality. Among the 17 patients who received IV dantrolene perioperatively, nine were confirmed to have MH. Notably, four of these patients had previously undergone uneventful general anesthesia with volatile agents, and all four tested positive for an *RYR1* variant associated with MH susceptibility. This observation highlights that a history of uneventful exposure to triggering agents does not negate the risk of MH susceptibility. The estimated prevalence of MH within our surgical population was 1 in 98,347 general anesthetics, consistent with previous reports [5, 6].

### Characterizing patients who received IV dantrolene: Insights into MH and MH-like events

**Figure 1.**
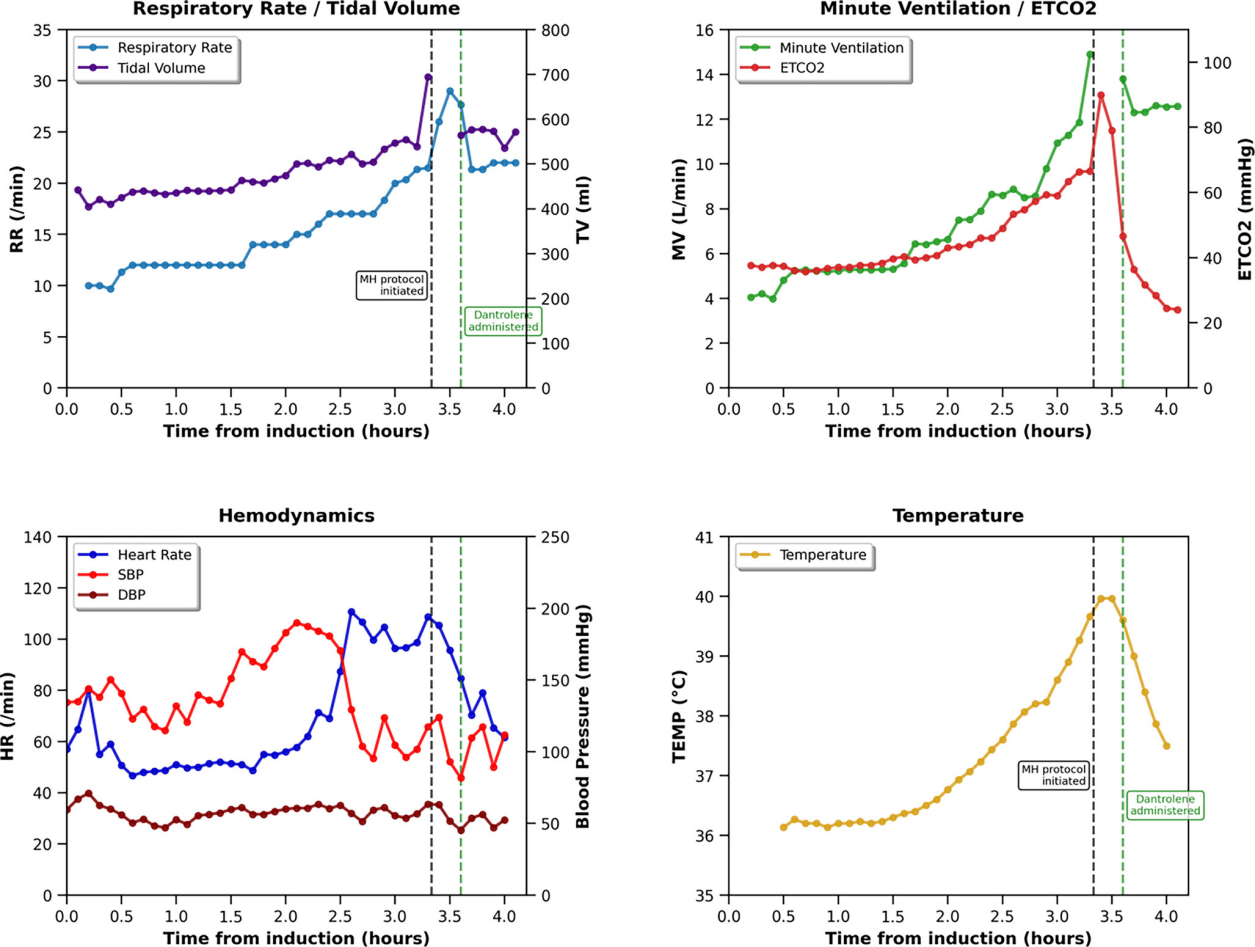
**Intraoperative hemodynamic, respiratory, and temperature changes in a 60-year-old man undergoing carotid endarterectomy.** This graphical representation depicts the gradual onset of malignant hyperthermia (MH) during anesthesia. An early intraoperative indicator of MH, particularly the rising end-tidal carbon dioxide (EtCO_2_), was initially obscured as the increasing EtCO_2_ levels were mitigated by augmenting minute ventilation. A full-blown MH crisis became evident only toward the conclusion of the procedure, characterized by severe hypercapnia (EtCO_2_ 100 mmHg, PaCO_2_ 88 mmHg), respiratory acidosis (pH 7.14), hyperkalemia (serum potassium 6.0 mmol/L), and hyperthermia (temperature 40.8 ^∘^C). This was followed by hemodynamic instability, transitioning from hypertension and tachycardia to hypotension. At that point MH was suspected, prompting the cessation of triggering agents, intravenous administration of dantrolene, and the initiation of active cooling measures. Both EtCO_2_ and temperature normalized within 30 min following treatment.

Among the clinically confirmed MH cases, five exhibited classic features, including an intraoperative rise in EtCO_2_ and other hypermetabolic signs. In anesthetized patients, a rising EtCO_2_ is often the earliest and most sensitive clinical indicator of MH (see Figure 1). However, MH can present atypically. Notably, one case involved a 3-month-old infant who developed severe hypercapnia and acidosis minutes after anesthesia induction, without fever or hyperkalemia. This observation aligns with reports from the Malignant Hyperthermia Association of the United States (MHAUS) Registry [7], which indicate that younger patients are less likely to exhibit fever and hyperkalemia as part of the MH complex. Another patient displayed MH-like symptoms during emergence from anesthesia, initially attributed to serotonin syndrome; however, due to persistent fever, dantrolene was administered empirically, resulting in fever resolution. Subsequent genetic testing confirmed susceptibility to MH. In the operative setting, a progressive and gradual rise in EtCO_2_, particularly when repeated ventilatory adjustments (increases in tidal volume and/or respiratory rate) are necessary to maintain normocapnia, should raise early suspicion for MH. However, MH may not be recognized until severe clinical signs become apparent (see Figure 1 and Figure S1). These cases underscore the importance of maintaining a high index of suspicion for MH, even when clinical signs are subtle, atypical, or occur outside the intraoperative period.

A subset of our patients received IV dantrolene for suspected NMS or other refractory febrile conditions (see Table S1). Although dantrolene is not considered first-line therapy for NMS, its administration may be warranted in cases complicated by significant muscle rigidity and markedly elevated creatine kinase levels. Among the nine (eight treated with IV and one with PO dantrolene) patients ultimately diagnosed with NMS, eight presented with fever, and all had elevated creatine kinase levels. Additionally, one patient with refractory fever due to central dysautonomia following neurotrauma was treated with IV dantrolene, resulting in fever resolution. Moreover, four additional patients received IV dantrolene for muscle rigidity associated with other disorders.

### The role of genetic variants in shaping MH phenotypic expression

Four of our patients were identified as carriers of *RYR1* gene variants associated with susceptibility to MH. All were heterozygous, which may partially account for their relatively mild clinical presentations. It is well established that the severity of *RYR1*-related myopathy is influenced by the specific genetic variant, demonstrating a clear genotype-phenotype correlation [8–12]. Patients with heterozygous *RYR1* mutations are generally anticipated to exhibit milder clinical features compared to those with homozygous mutations [13]. However, the interpretation of genetic testing remains complex, as the majority of reported MH cases involve heterozygous *RYR1* variants [8, 14]. Homozygous variants have been documented in two patients with the Cys35Arg substitution and in one patient with the Arg614Cys substitution [11, 14, 15]. Regardless of zygosity, any patient testing positive for an MH-associated gene variant should be managed with heightened perioperative caution.

### Using dantrolene administration as a surrogate marker for MH prevalence

Research into the incidence or prevalence of rare diseases, such as MH, presents significant methodological challenges. In response, collaborative registries involving government health agencies, academia, industry, and advocacy groups, such as MHAUS [7], have been established to gather data from affected individuals. Automated analysis of large electronic healthcare databases offers a complementary approach to estimating the frequency of rare disorders [16–18]. In our cohort, perioperative IV dantrolene use was associated with an MH diagnosis in 53% of cases exhibiting MH-like symptomatology, supporting its potential utility as a marker for identifying patients with clinical presentations consistent with MH. Estimating the prevalence of adverse clinical events based on the administration of event-specific medications has precedent in perioperative research. For instance, postoperative naloxone administration has been utilized as a surrogate marker for opioid-induced respiratory depression [19], while the use of rescue antiemetics has served as an indicator for the incidence of postoperative nausea and vomiting [20]. However, unlike the aforementioned cases, dantrolene is not exclusive to MH; it has multiple clinical indications. Thus, employing dantrolene administration as a surrogate marker for MH necessitates a thorough review of medical records to ascertain the specific clinical context, including exposure to known triggering anesthetics and the rationale for its use. Furthermore, relying on IV dantrolene administration as an indicator of emerging MH likely has low sensitivity and may overlook mild or atypical cases. For example, we recently encountered a patient who developed mild masseter spasm during anesthesia induction with propofol and succinylcholine [21]. The anesthetic course under total IV anesthesia was unremarkable; however, this patient subsequently developed severe rhabdomyolysis immediately postoperatively, exhibiting no other MH-related signs and not receiving dantrolene. Whole-genome sequencing later revealed a heterozygous variant in the *RYR1* gene (c.1840C>T), consistent with MH susceptibility. This case underscores the possibility that atypical or mild MH presentations may remain unrecognized and untreated, leading to an underestimation of MH prevalence when dantrolene administration is used as a surrogate marker for MH diagnosis [18].

### Limitations

This study is subject to the inherent limitations of a retrospective design. MH is a potentially elusive disorder with a wide spectrum of clinical presentations, ranging from mild to fulminant and from typical to atypical manifestations, complicating early recognition and treatment. Notably, milder or atypical episodes of MH may go unrecognized and are likely managed without dantrolene, resulting in an underestimation of MH prevalence when using dantrolene administration as a proxy for MH episodes. Additionally, since MH cases were identified based on dantrolene use, there is a possibility that ambulatory surgical patients may have experienced MH after hospital discharge. However, it is likely that patients with severe reactions would have required prompt readmission, in which case they would be documented through our pharmacy records. Nevertheless, there remains the possibility that patients with mild clinical presentations did not seek further medical attention or did so at another facility, thus not being captured in our dataset.

## Conclusion

Dantrolene use was infrequent in our hospitalized population. Oral doses were primarily prescribed for chronic spasticity, while perioperative IV administration was more commonly linked to suspected MH. However, specificity was low, as dantrolene was also administered for conditions such as NMS or serotonin syndrome. The estimated MH prevalence was approximately 1 in 100,000 anesthetic exposures, although this figure is likely an underestimate, as mild or atypical cases may go unrecognized. Our findings also reaffirm that a history of uneventful anesthesia does not exclude the possibility of MH susceptibility.

## Supplemental data

Supplemental data are available at the following link: https://www.bjbms.org/ojs/index.php/bjbms/article/view/13340/4047.
